# Herb-Induced Liver Injuries in Developing Nations: An Update

**DOI:** 10.3390/toxics6020024

**Published:** 2018-04-17

**Authors:** Cecilia Nwadiuto Amadi, Orish Ebere Orisakwe

**Affiliations:** Department of Experimental Pharmacology & Toxicology, Faculty of Pharmacy, University of Port-Harcourt, PMB, 5323 Port Harcourt, Rivers State, Nigeria; cnamadi@rocketmail.com

**Keywords:** liver disease, risk assessment, public health, herbal, herbs and dietary supplements

## Abstract

The last few decades have seen a rise in the use of herbal supplements, natural products, and traditional medicines. However, there are growing concerns related to the safety and toxicities of these medicines. These herbal medicines are associated with complications such as liver damage with a high incidence of mortalities and morbidities. Clinical manifestations range from asymptomatic cases with abnormal liver functions tests to sudden and severe liver failure necessitating liver transplantation. This work aimed to review the etiology, risk factors, diagnosis, clinical manifestations and selected clinical case reports of herbal hepatotoxicity in developing nations. PubMed and Google Scholar searches were undertaken to identify relevant literature. Furthermore, we scanned the reference lists of the primary and review articles to identify publications not retrieved by electronic searches. Little data exists on clinical cases of herb-induced liver injury in some developing countries such as Nigeria, as most incidences are either not reported to health care providers or reports from hospitals go unpublished. Studies in Nigeria have highlighted a possible correlation between use of herbs and liver disease. In Uganda, and association between the use of traditional herbal medicine with liver fibrosis in HIV-infected and non-HIV patients was demonstrated. Reports from China have revealed incidences of acute liver failure as a result of herbal medicine use. The actual incidence and prevalence of HILI in developing nations remain largely unknown due to both poor pharmacovigilance programs and non-application of emerging technologies. Improving education and public awareness of the potential risks of herbals and herbal products is desirable to ensure that suspected adverse effects are formally reported. There is need for stricter regulations and pre-clinical studies necessary for efficacy and safety.

## 1. Introduction

The liver is one of the vital organs in the human body, and is highly susceptible to a wide array of metabolic, toxic, microbial, circulatory, and neoplastic injury. Common liver diseases include: viral hepatitis, inflammatory diseases, alcoholic liver disease, non-alcoholic fatty liver disease (NAFLD), and hepatocellular carcinoma [1,2]. In most cases, liver diseases start as a gradual and subtle process in which clinical detection and manifestation could occur weeks, months, or even many years following onset of injury. Therefore, at the point at which most patients with hepatic dysfunction are referred to hepatologists, they already have chronic liver disease [3].

Liver injury associated with the consumption of herbal medicines is referred to as ‘herb-induced liver injury’ (HILI), which occurs rarely in only a few susceptible individuals [4,5]. The clinical manifestations of HILI are identical to those of drug-induced liver injury (DILI) [4]. Furthermore, HILI and DILI share common features, as both cases are caused by chemical components that can be produced either by natural or synthetic processes. These natural and synthetic chemicals are foreign to the body and require metabolic breakdown to be eliminated. However, in the course of metabolism, substances that are toxic to the kidney could be produced, resulting in liver injury in susceptible individuals [6].

It is important to note that HILI is often self-limiting; however, continual liver damage, acute liver failure (ALF), death, and liver transplantation have been indicated [7]. Herb/drug-induced liver injury have characteristics comparable to those other liver diseases unrelated to herbs and drugs [8,9]. The use of herbal medicines in developing nations has risen in recent times. It is estimated that over three-quarters of the population in sub-Saharan Africa depends on traditional herbal remedies for primary health care [10]. For example, in Nigeria, common herbal preparations are obtained from green water leaves, bark of *Mangifera indica* (mango), shoot of *Anacardium occidentale* (cashew) leaves, *Carica papaya* (paw-paw) leaves, lime, *Solanum erianthum* (potato tree), and *Azadirachta indica* (Neem) tree [11].

It is also known that complementary and alternative medicines have gained popularity in the general population, even among patients and physicians. Patients believe these products are natural in origin, and hence safe for consumption. In contrast to this belief, the literature has proven this to be untrue, with multiple reports of hepatotoxicity [12,13]. Interestingly, some plants produce toxic compounds as secondary metabolites, which may not easily be distinguishable from the active pharmacological constituents. Some of these herbs are produced in very unhygienic conditions using potentially toxic ingredients, subsequently exposing the consumers to multiple hepatotoxins [14]. It is also worthy of note that some marketed herbal products are composed of complex mixtures, hence the exact component that is responsible for injury is difficult to discern.

Herbal medicines have been implicated in herb-induced liver injury (HILI) in sub-Saharan Africa. Minimal data exist on the hepatotoxicity of commonly used herbs or the contribution of herbs to the burden of liver disease in sub-Saharan Africa. The incidence of HILI is more difficult to document than DILI, because of the use of a wide variety of non-commercial and non-prescribed herbal medicines or dietary supplements. Furthermore, the incidence of HILI is underestimated because of the low frequency with which patients report their use. Presently, there is a growing concern on the potential risks of HILI from herbal products in sub-Saharan Africa, because the contents of most of these medicines are unregulated and unstandardized. These herbal products include herbal medicines, health foods and dietary supplements whose widespread use have become a global problem. Despite the widespread belief that herbs and herbal products are of natural origin and, unlike western medicines, are to be considered safe and are without many side effects, there have been many reports of adverse effects linked with herbal remedies [15]. Whereas there is documented evidence of herb-induced liver injury (HILI) in developed countries based on robust pharmacovigilance programs, information is sparse for the data-poor communities of sub-Saharan, Asian and Caribbean countries. The present review is an updated synoptic capture of herb-induced liver injury in developing nations where patronage of herbal dietary supplements is very high.

## 2. Methods 

### 2.1. Database Searching, Search Strategy and Selection Criteria

PubMed and Google Scholar searches were undertaken up until August 2017, to identify relevant literature using search terms including ‘herbal’, ‘herbs’, ‘dietary supplement’, ‘liver injury’, ‘herb-induced liver injury’, ‘drug-induced liver injury’, ‘hepatitis’ and ‘drug hepatotoxicity’. Furthermore, we scanned the reference lists of primary and review articles to identify publications not retrieved by electronic searches. Search results were screened, and all titles and abstracts were read for eligibility. For any potentially eligible articles, full texts were obtained and inclusion and exclusion criteria were applied to determine the suitability of the article to be included within the review. The criteria used to assess the quality of the studies were the same as those proposed by AMSTAR [16].

### 2.2. Inclusion Criteria and Exclusion Criteria 

The inclusion and exclusion criteria were applied when reviewing the title and abstract of each article for this review. Studies were included if they reported hepatotoxicity due to herbs, herbal drugs and herbal dietary supplements. Also included were articles that reported original data and studies conducted in the human population. Articles were excluded if adverse herb events were not associated with liver toxicity, and if the research articles were not available in English. If more than one report was published from the same study, the most recent study was included. No limits were applied to the year of study.

## 3. Current Status of Knowledge 

### 3.1. Results and Discussion

#### Results of Search

The initial search found a combined total of 92 studies. After screening titles and abstracts, we excluded 13 articles, leaving 79 articles for full text review. These articles were excluded on the basis of being irrelevant (*n* = 9), no copy available in English (*n* = 1), and duplicates (*n* = 3). 

**Figure 1:**
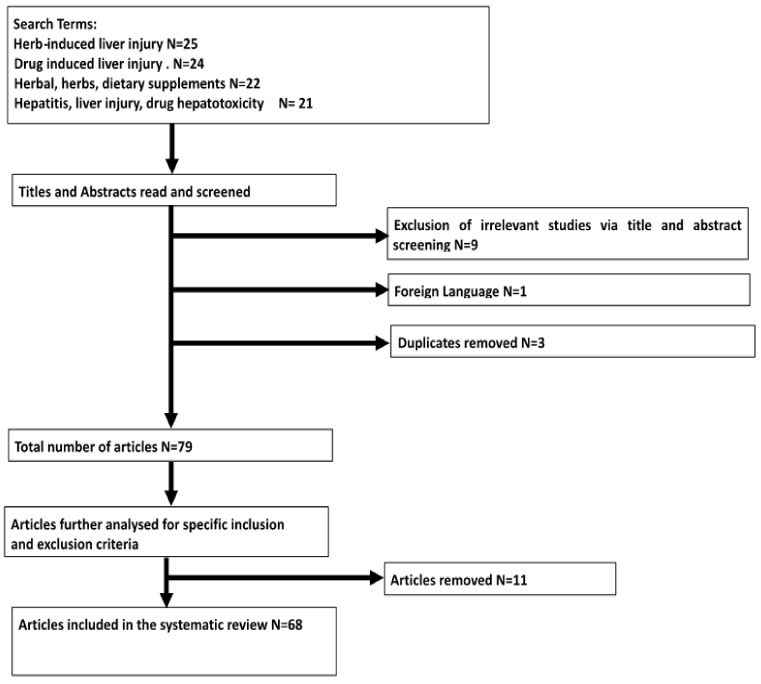
Study selection flow diagram.

We excluded 11 further articles after full text review to ascertain the suitability of the articles. After the inclusion and exclusion criteria were applied, 68 articles were included in this review, as shown in Table 1 and Table 2. The process is summarized in Figure 1.

### 3.2. Etiology and Risk Factors Associated with the Development of HILI 

In Nigeria, herbal remedies are usually recommended for malaria, typhoid, diabetes, fever and infectious diseases [17]. Most adverse effects associated with the use of herbal medicines are not reported to the regulatory bodies or national pharmacovigilance centers, which is indicative of inadequate monitoring of adverse effects (Pharmacovigilance) [17]. It is also a known fact that herbs and herbal products (herbal mixtures inclusive), contain multiple active constituents rather than one single constituent, as obtained with synthetic drugs [9].

Hepatotoxicity from herbal medicines can result from any of the following: (i) use of herbs with unknown toxicity; (ii) incorrect identification leading to substitution of an non-poisonous herb with a toxic one; (iii) deliberate or inadvertent contamination with hepatotoxic non-herbal drugs (e.g., non-steroidal anti-inflammatory agents), pesticides, chemicals and heavy metals; and (iv) potentiation of the toxic effect of a conventional drug due to interaction with a compound present in the herbal preparation, e.g., alcohol [9,18]. Therefore, problems of herbs and herbal hepatotoxicity could be linked to production factors, i.e., the quality of herbal products, risks of impurities, contaminants and adulterants, incorrect use of plant species and plant parts, patient factors, e.g., co-medication, co-morbidity, self-medication without disclosure to physicians, and incorrect diagnoses [18]. 

#### 3.2.1. Incorrect Identification and Mislabeling of Herbal Products

Correct information of the individual herb composition is best provided by the manufacturers in the product leaflet, which is available to doctors who suspect HILI. Herbal product misidentification remains hidden for consumers who are of the belief that the product they ingest actually contains the correct herbal components as indicated on the label. Herbal products containing incorrectly identified plants pose a high risk for the unassuming consumer. Mislabeling of herbal products could result in incorrect causality attribution until clarification of composing ingredients is made using standard product analysis [6]. Mislabeling has been identified in some herbal remedies after product analysis and result comparison with the product labeling [19]. It has been reported that many herbal products commonly contain green tea extract (GTE) and its component catechins, which are implicated in hepatotoxicity, but their presence may not be clearly indicated on the product label [19]. This implies that for a patient with suspected HILI, confirmations may not be made without careful product analysis. This, however, requires that the offending herbal product be subjected to analysis and adequate testing [6]. A notable example of mislabeling is highlighted by a hepatotoxicity case from Hong Kong, in which hepatic sinusoidal obstruction syndrome (HSOS) was associated with *Sedum aizoon*. However, this turned out to have been caused by another traditional Chinese herbal medicine—Shan Chi (*Gynura segetum*) [20]. 

#### 3.2.2. Co-Morbidities and Drug Interactions

Co-morbid conditions could warrant concomitant administration of certain drugs with herbs, leading to further exaggeration of hepatotoxic effects of drugs. For example, pre-existing liver disease has been identified as an important other risk factor. This has been observed in patients with viral hepatitis and tuberculosis co-infections who develop liver injuries as a result of antiviral and antituberculosis drugs [21].

Herbs may enhance anti-retroviral therapy (ART) toxicities by affecting antiretroviral drug metabolism in the liver and gut [22]. Herbs may affect drug metabolism by CPY3A4 and impair the activity of cellular drug transporters and glucuronidation pathways [22]. It is reported that certain herbs cause a dose-dependent inhibition of CYP3A4 and reduction of the expression of P-glycoprotein, leading to drug accumulation and exaggeration of hepatotoxicity of co-administered drugs [21]. In some HILI incidences, co-medication with herbs and synthetic drugs is common, predisposing a potential drug-herb interaction at the hepatic cytochrome P450 (CYP) system [9,23]. Herb–drug interactions have been attributed a wide spectrum of adverse reactions. Herb–drug interactions may alter specific receptor number or affinity or result in pharmacokinetic changes by impairing absorption, distribution, metabolism and excretion [24].

#### 3.2.3. Alcohol Consumption 

The number of heavy episodic drinkers has risen in West Africa in recent times [25], and heavy alcohol consumption has been shown to be a risk factor for liver injury, since alcohol is a known hepatotoxin on its own [7]. Alcohol is used as a vehicle and solvent in most herbal medicines, predisposing patients to multiple hepatotoxins at a time [14]. Alcohol activates enzymes that could transform certain drugs (e.g., acetaminophen) into more toxic compounds that can be injurious to the liver [26]. Alcohol may impair liver function by causing liver cirrhosis and liver hepatitis, which subsequently affects its drug metabolizing capacity [26]. For example, a study in Nigeria by Ndububa et al. has indicated alcohol consumption to be an independent determinant of progression of chronic liver disease [27]. Furthermore, in Jos, Nigeria, alcohol was shown to be the cause of liver cirrhosis in about 80% of patients, as obtained in a clinical investigation [28]. Another study by Navarro et al. [29] demonstrated that alcohol consumption predisposes humans to toxic effects of body-building supplements as compared to other medications.

#### 3.2.4. Adulterants, Impurities and Contaminants 

Heavy metals such as lead, mercury, cadmium, or arsenic are sometimes found in some herbal medicines, added as adulterants in the belief that they could enhance the effectiveness of the herbal products [6]. Some of these toxic metals are known to be hepatotoxic, and toxicity of medicinal plants is often times associated with environmental sources of the plants. Considering the increased consumption of herbal remedies in developing nations, especially Nigeria, and the discharge of industrial waste on the surrounding vegetation, heavy metal contamination in some commonly used medicinal plants is quite inevitable. A study done in Nigeria by Awodele and coworkers investigating the heavy metal contents of some traditional medicine plants demonstrated significantly higher concentrations of Lead (Pb), Cadmium (Cd), Chromium (Cr), Nickel (Ni) and Zinc (Zn) in the leaves and roots of plants harvested from polluted soil than those collected from unpolluted soil. Heavy metal levels were also significantly higher in polluted than in unpolluted soil samples [30]. Earlier studies in Nigeria also demonstrated elevated levels of cadmium, copper, iron, nickel, selenium, zinc, lead and mercury in random samples of traditional remedies. Data reported showed that 100% of the samples contained elevated levels of these heavy metals [31]. This is consistent with work done by Amadi et al., which evaluated heavy metal contamination of registered herbal supplements in Nigeria. Results from this study revealed high concentrations of mercury, antimony and tin in most of the samples [32].

Synthetic drugs have also been indicated as common adulterants in herbal products. These synthetic compounds are added to some herbal products to possibly enhance therapeutic effects, but sometimes the synthetic drug adulterants are not labeled as components of these herbal products [6,9]. For instance, some Chinese herbal medicines have been reported to contain synthetic drug contaminants [33]. Hence, a question arises as to whether the liver disease is a HILI by the ingested herbs or a DILI caused by the synthetic drug. 

To guarantee maximal agricultural yield, farmers apply various types of pesticides to control pests and diseases in plants. Organophosphate pesticides are commonly utilized in agriculture for the purpose of pest control in developing countries. For example, dichlorvos, organophosphate pesticide primarily acts by irreversibly inhibiting acetyl cholinesterase enzyme (AChE) at cholinergic junctions of the nervous system and produces hepatotoxicity in rats [34]. A recent study done in Lusaka, Zambia, evaluating residual levels of dichlorvos in vegetables revealed that the average levels of dichlorvos were significantly higher than the maximum accepted limit set by the Zambian Food and Drugs Act for vegetables [35]. 

Furthermore, other microbial contaminants like aflatoxin have also recently been identified in herbal products. Aflatoxin is a potent hepatotoxin and hepatocarcinogen [36]. Aflatoxin contaminants evolve from the agricultural and manufacturing processes of herbal medicines as a result of humid conditions and high temperatures [6]. In West Africa, high exposures to aflatoxins are reported due to poor processing and long-term storage of crops enhancing the growth of *Aspergillus* spp. [37,38]. Scientific work investigating the microbial load and aflatoxin levels in herbal medicines from selected states in Nigeria was carried out recently by Ezekwesili-Ofili and coworkers [39]. Aflatoxin B1, B2 and G1 were detected in in the samples analyzed, with an average occurrence of 18.6%. Some of these herbal remedies were shown to contain an unacceptably high bioload, which exceeded WHO standards [39].

#### 3.2.5. Host-Related Risk Factors

Host-related risk factors for drug-induced hepatotoxicity include age, malnutrition, and sex [40]. It is widely believed that liver injuries due to drug toxicities occur more frequently in adults as compared to children [41]. Recent reports have further highlighted that the incidence of liver injuries is higher in patients aged over 40 years and also increases with age [7,42,43]. This could be due to the fact that adults are more commonly exposed to potential hepatotoxins than children. Females have also been indicated to be at higher risk than males [44]. For certain drugs, genetic variations appear to predispose certain individuals to the risk of developing liver injuries, e.g., isoniazid DILI [45].

### 3.3. Diagnosis and Clinical Manifestations of HILI

The diagnosis of hepatotoxicity due to herbs and herbal products is usually made in the same way as for conventional drugs. However, patients must often be persuaded into revealing a history of use. Diagnosis of liver injury starts with a history of the ingested herbal product and exclusion of other causes of injury, such as viral hepatitis, autoimmune disease, anatomic malformations, and metabolic anomalies [46,47]. The diagnosis of herb-induced liver injury HILI is difficult because none of the conventional assessment methods accurately assesses hepatotoxicity associated with herbs [46]. Nevertheless, physical examination, liver function tests, and differential diagnoses are required [48], and discontinuation of herbal use is highly recommended if HILI is suspected following diagnosis. 

Liver enzymes are important biomarkers of the degree of liver damage and are easily available for the monitoring of patients with liver disease in developing countries, where the menace of this disease is high, with inadequate invasive diagnostic facilities [49]. Elevated alanine aminotransferase (ALT) levels are the most frequently used indicator for chronic liver disease. Even though it is produced by other organs, it is found mainly in hepatocytes and is thus considered a specific marker for liver injury [50,51]. 

Liver injury has a wide array of clinical manifestations, ranging from asymptomatic mild biochemical irregularities to severe hepatitis with jaundice. In most cases, liver injury accruing from drug/herb use improves after discontinuation of the suspected agent [52]. Clinical jaundice due to administered agent has been correlated with a fatality rate of 10% for numerous drugs [53]. It has also been reported that elevation of the activities of transaminase enzymes in combination with jaundice indicates critical liver injury with fatalities [53,54,55,56]. These findings have been highlighted as Hy’s law for monitoring liver injuries, which states that elevation of liver enzymes (AST or ALT more than 3× ULN (upper limit of normal) or ALP more than 1.5× ULN) in addition to increased bilirubin levels (more than 3× ULN) at any time after administration of a new drug could indicate serious liver injury. Hence, it is advised that treatment with the suspected drug be discontinued following such manifestations [57,58]. However, a recent work has revealed that cases fulfilling Hy’s rule have not often resulted in death from DILI [59]. It was observed that many drugs can induce an asymptomatic increase in liver enzymes without severe hepatotoxicity. With this known fact, the Food and Drug Administration (FDA) produced guidelines indicating that ALT greater than 8× ULN, ALT greater than 5× ULN for two weeks, ALT greater than 3× ULN in addition to serum bilirubin greater than 2× ULN, more than 1.5× PT-INR, or symptoms of liver injury be used to diagnose severe hepatotoxicity with discontinuation of the offending drug [60]. 

On the other hand, the CIOMS criteria regarding DILI categorizes liver injury as (a) ALT > 2× ULN (upper limit of normal range), (b) DB > 2× ULN, or (c) concurrent elevation in AST, ALP, and TB, with one value > 2× ULN [61]. Hence, hepatic injuries can be classified into as hepatocellular, cholestatic, or mixed types of injury according to the *R* ratio, with causality assessment being done using the *R*-score by means of the Roussel Uclaf Causality Assessment Method (RUCAM), where the R ratio is (ALT/ ULN of ALT) to (ALP/ULN of ALP). For Hepatocellular injury: *R* ≥ 5, or (ALT > 2× ULN and ALP in normal range), for cholestatic injury: *R* ≤ 2, or (ALP > 2× ULN and ALT in normal range) and for mixed injury: 2 ≤ *R* < 5 and (ALT > 2× ULN and ALP > ULN) [61,62].

### 3.4. Selected Clinical Case Reports of DILI in Sub-Saharan Africa and Other Developing Nations

Little data exists on clinical cases of drug/herb-induced liver injury (DILI/HILI) in developing nations, as most cases are either not reported to health care providers by the patients, or reports from hospitals remain unpublished. Table 1 and Table 2 show selected case reports of DILI and HILI in sub-Saharan Africa and other developing nations. 

In India, a study was performed by Devarbhavi et al. between 1997 and 2008 to characterize the causes, outcomes, predictors, and models for 90-day mortality from DILI [63]. Out of the 313 patients enrolled in the study, 58% were males. The major causes of DILI were a combination of four anti-tuberculous medications (58%), anti-epileptic medications (11%), olanzapine (5%), and dapsone (5%). Mortality (17%) was significantly higher for hepatitis resulting from anti-tuberculous drugs (22%). The highest mortality was observed from leflunomide (75%). Severe hepatic failure developed more in females than in male patients (23% vs. 17%). Sixty-six percent (66%) of jaundice and/or icterus cases were observed, with 26% mortality [63]. More recent research from India also reported acute liver failure in a quarter of DILI patients who received antituberculosis treatment, and the overall mortality was shown to be about 23% [66]. Data from China also indicated that anti-tuberculosis drugs caused 32% of the mortalities in DILI, which is indicative of the hepatotoxicity of anti-tuberculosis drugs [43]. These results are also consistent with investigations carried out in Jos, Nigeria to evaluate the hepatotoxicity of anti-tuberculous drugs in 110 hospitalized patients, which revealed manifestations of hepatotoxicity in twenty patients after a period of six months [64]. 

### 3.5. Selected Clinical Case Reports of HILI in Sub-Saharan Africa and Other Developing Nations

A study carried out by Nwokediuko and group probed the pattern of liver disease admissions in a Nigerian tertiary hospital [14]. The results obtained revealed that while liver diseases accounted for 8% of medical admissions, ingestion of herbs and roots were a factor in 46% of the cases [14]. Their work demonstrated a possible correlation between consumption of herbs and liver disease. In Uganda, research was carried out by Auerbach and coworkers to investigate the association between the use of traditional herbal medicines for liver fibrosis in HIV-infected and non-HIV patients [21]. Participants were probed on traditional herbal medicine use and other potential risk factors for liver disease. The results obtained demonstrated a significant correlation between traditional herbal medicine use and an increase in significant liver fibrosis in both HIV-infected and HIV-uninfected study groups [21]. 

Reports from China by Zhao et al. identified 30 patients (6 men and 24 women) who had acute liver failure as a result of herbal medicine use. Out of these 30 patients, 18 died without receiving liver transplantation [67]. Another dataset processed by Ou et al. from between 2011 and 2014 in China also identified Chinese herbal medicine as the primary cause of liver injury in 36% of the patients investigated in their study [43]. This is consistent with data obtained from Shanghai (China), which further confirmed that Chinese herbal medicine accounted for 54% of liver injuries cases in hospitalized patients [68]. Earlier studies in 2006 described the clinical course of twenty-nine patients with liver injury at a tertiary liver center in Singapore over a 12-month period. Sixty-nine percent (69%) of these patients were female, and the median age was 51 years (range 18–76) and 83% were Chinese. Traditional Chinese medicines (TCM) were mostly implicated, as 52% of the patients presented liver injury from TCM, while 14% were from anti-tuberculosis medications. Sixty-two percent (62%) of the patients presented with hepatitis, 24 percent presented with cholestatis and 14% had mixed symptoms. Extrahepatic symptoms were observed in only 10% of the patients, and another 10% died, whereas 3% received liver transplant for liver failure [65]. Further to this, in 2007, Wai et al. described a prospective study over a 26-month period to study HILI in Asia and to check whether liver injury caused by traditional complementary and alternative medicine (CAM) was related to adulterants [69]. Thirty-one patients (18–79 years) with HILI were investigated (17 male and 14 female patients). Twenty-three (74%) were found with hepatocellular injury, six (19%) had cholestatic, and two (7%) had a mixed pattern of injury. Interestingly, Chinese traditional medicine was the most common herbal medication type involved in injury, accounting for seventeen (55%) patients, followed by Malay CAM in five (16%) patients. Adulterants were also found in nine (29%) of the herbal products following chemical analysis [69]. Similarly, severe hepatotoxicity was reported from prolonged ingestion of Indian Ayurvedic herbal products used for vitiligo. However, a rapid symptomatic improvement was noted after discontinuation of herbal product [70]. These studies indicate herbs and herbal medicines as important causes of liver injuries in developing nations.

### 3.6. Challenges of HILI Management in Sub-Saharan Africa 

The use of herbs and herbal products is grossly under-reported in sub-Saharan Africa. It is documented that approximately half of users of herbal products fail to report the use of these products to their health-care providers and sometimes, even when they do so, fail to disclose them all [71].

In Nigeria, studies have revealed that inadequate monitoring of adverse effects underscores the need to educate and enlighten herbal medicine practitioners on the need for pharmacovigilance activity of herbal products [17]. Paradoxically, physician knowledge on the clinical use and safety profile of herbs and herbal products is poor, and this does not encourage an open dialogue on the use of these medications with patients [72]. Hence, this inadequate knowledge also leads to under-reporting of herb-related adverse events. Reports have indicated that, about three in four doctors do not know how or where to report drug-related adverse events [73]. Lack of confidence on the available health infrastructure on the part of the patient, contributes to poor health-seeking attitudes and this culminates to poor management of the disease. Unavailability of resources, health infrastructure, and high cost of treatment are important contributory factors, as well [74]. In addition, incorrect or missed diagnosis in suspected HILI cases is a major cause for concern, because delayed administration of adequate therapy is associated with the risk of prolonged or permanent health hazards [9].

The dosages of herbal drugs and their compositions vary between traditional medicine practitioners, and it is thus very difficult to identify which component or, rather, components is/are the cause of the liver injury. Little is known about components of herbal remedies that could potentially cause liver injury. This brings about an urgent need to standardize the compositions of herbal medication and provide guidelines for dosage.

## 4. Conclusions

Herbal medicines have been indicated as a major cause of liver injuries [44,75]. Interestingly, the use of these medicines continues to rise in developing countries, especially amongst the rural population. Lack of regulation is a major factor behind the widespread use of potentially toxic herbs. The exact incidence and prevalence of HILI due to hepatotoxic herbs remains uncertain. Although underreporting, poor record keeping, and weak pharmacovigilance programs have contributed to the rather lean size of literature on HILI in developing nations, this study, by thorough examination of the literature, has confirmed a magnitude of HILI that is deserving of public health attention. 

The clinical manifestations of HILI range from asymptomatic or abnormal hepatic biochemical tests to acute liver failure requiring a liver transplant [48]. Sub-Saharan Africa is made up of 47 countries with over 900 million people, and the gross domestic product per person is below US$1500 per year on average [76]. Liver transplantation is, however, very expensive and is unaffordable to a good proportion of the population in sub-Saharan Africa. In view of the high morbidity and mortality from HILI and the high cost of treatment/management, its prevention is the best option. We therefore propose that stricter reporting of herb-induced liver injury to health care institutions and optimal evaluation of the mechanisms of hepatotoxicity from these compounds would help curb the serious outcomes associated with usage. 

What is already known on this topic:

□ There is increasing incidence of liver disease in developing nations.
□ Herbal medicines are associated with complications such as liver damage with a high incidence of mortalities and morbidities.

What this study adds:

□ Poor pharmacovigilance programs in sub-Saharan Africa and other developing nations remain a challenge in the documentation of herb-induced liver injury (HILI).
□ Although this study has attempted to capture HILI in developing nations, it is feared that the present data is a far cry from the reality, given the increasing incidence of liver disease.
□ There should be an introduction of sound regulatory policies predicated on contemporary science and emerging technologies to boost predictive and preventive medicine in developing nations.

## Figures and Tables

**Table 1:** Selected clinical case reports of DILI in developing nations.

Countries and Patient Characteristics	Clinical Cases and Prognosis	Reference
India (1997–2008), *N* = 313; liver injury, Single-centre, retrospective	Liver injury resulted in 17% overall mortality	[63]
Nigeria (January to June, 2013), *N* = 110, liver injury, single centre, retrospective	Results revealed symptomatic hepatotoxicity in twenty patients with an incidence of 18%	[64]
Singapore (2003–2004), *N* = 29, liver injury, single centre, prospective	Fifteen patients (52%) had liver injury from TCM, while four patients (14%) had liver injury from anti-tuberculosis drugs. Eighteen patients (62%) had hepatitis, seven patients (24%) had cholestatic injury, and four patients (14%) had mixed injury. Three patients (10%) died and one patient (3%) had liver transplant for liver failure. Chinese herbal medicine was majorly implicated	[65]

**Table 2:** Selected clinical case reports of HILI in developing nations.

Countries and Patient Characteristics	Clinical Cases and Prognosis	Reference
Nigeria (2005–2010), *N* = 365, HILI, Single centre, retrospective	Consumption of herbs and roots was indicated as a risk factor in 46% of patients with liver diseases	[14]
Uganda(1994–1998), 500 HIV-infected and 500 HIV-uninfected participants, Single centre, HILI	For all participants, use of herbs was associated with significant liver fibrosis	[21]
China (2011–2014), *N* = 469, liver injury, Single centre, retrospective	The incidence rate of liver injury was 93 cases per 100,0000 patients. Chinese herbal medicine was highlighted as the major cause of liver injury in 36% of patients	[43]
China (2007–2012), *N* = 30, HILI, Multi-centre, retrospective	Acute liver failure with 60% mortality (18 patients died). Chinese medicinal herbs were implicated	[67]
China (2008–2010), *N* = 138, liver injury, Single centre, retrospective	Chinese herbal medicine was the major cause of liver injury resulting 54% of cases. Higher incidences of inflammation and fibrosis in cholestatic and mixed injury types than in the hepatocellular type	[68]
Singapore (2004–2006), *N* = 31; liver injury, Single centre, prospective	Twenty-three patients (74%) had hepatocellular injury, six patients (19%) had cholestatic injury, and two patients (7%) had mixed injury. Chinese herbal medicine was majorly implicated	[69]
